# Pharmacological Potential of Three Berberine-Containing Plant Extracts Obtained from *Berberis vulgaris* L., *Mahonia aquifolium* (Pursh) Nutt., and *Phellodendron amurense* Rupr

**DOI:** 10.3390/biomedicines12061339

**Published:** 2024-06-17

**Authors:** Alexandra Ciorîță, Sabina-Emanuela Erhan, Maria Loredana Soran, Ildiko Lung, Augustin Catalin Mot, Sergiu Gabriel Macavei, Marcel Pârvu

**Affiliations:** 1 Faculty of Biology and Geology, Babes-Bolyai University, 44 Republicii, 400015 Cluj-Napoca, Romania; marcel.parvu@ubbcluj.ro; 2 National Institute for Research and Development of Isotopic and Molecular Technologies, 400293 Cluj-Napoca, Romania; loredana.soran@itim-cj.ro (M.L.S.); ildiko.lung@itim-cj.ro (I.L.); augustin.mot@ubbcluj.ro (A.C.M.); sergiu.macavei@itim-cj.ro (S.G.M.); 3 Faculty of Chemistry and Chemical Engineering, Babeș-Bolyai University, 11 Arany János St., 400028 Cluj-Napoca, Romania

**Keywords:** berberine, *Berberis vulgaris*, *Mahonia aquifolium*, *Phellodendron amurense*, spheroids, cytotoxicity, green synthesis, silver nanoparticles

## Abstract

Three berberine-containing plant extracts were investigated for their pharmacological properties. The stems and leaves of *Berberis vulgaris*, *Mahonia aquifolium*, and *Phellodendron amurense* were characterized through scanning electron microscopy. The plant extracts obtained from fresh stem barks were further analyzed through high-performance liquid chromatography, revealing berberine concentrations, among berbamine and palmatine. The plant extracts were further tested for their anticancer potential against 2D and 3D human skin melanoma (A375) and lung adenocarcinoma (A549) cell lines. The concentrations at which 50% of the cells are affected was determined by the viability assay and it was shown that *B. vulgaris*, the plant extract with the highest berberine concentration, is the most efficient inhibitor (0.4% extract concentration for the 2D model and 3.8% for the 3D model). The membrane integrity and nitrate/nitrite concentration assays were consistent with the viability results and showed effective anticancer potential. For further investigations, the *B. vulgaris* extract was used to obtain silver nanoparticles, which were characterized through transmission electron microscopy, energy dispersive spectroscopy, and X-ray diffraction. The formed nanoparticles have a uniform size distribution and are suited for future investigations in the field of biomedical applications, together with the *B. vulgaris* plant extract.

## 1. Introduction

Plant extracts are known for their antiviral, antibacterial, and antifungal properties due to their complex phytochemical composition [1]. The use of and interest in plant applications and consumption have increased in recent years, and this has led to more advances in this domain [2]. Recent studies demonstrate various beneficial effects of plant extracts and their natural compounds such as alkaloids. Berberine is an isoquinoline alkaloid found in several plant extracts and is known for its anti-tumor effect, by intensifying the apoptosis process of tumor cells, by modulating its activity, but also the activity of several pro-apoptotic and anti-apoptotic genes [3,4]. Other beneficial effects of extracts containing berberine are the antioxidant and anti-inflammatory properties [5,6].

*Berberis vulgaris* L. (barberry) belongs to Berberidaceae family and is found from central and southern Europe to western Asia and northwest Africa [7]. The fruit and root of *B. vulgaris* are also studied for their medical purposes [2], along with the stem and bark. The major bioactive constituents found in *B. vulgaris* are the isoquinoline alkaloids. Berbamine, tetrandrine, and chondocurine are protoberberines and bisbenzylisoquinoline alkaloids with anti-inflammatory and immunosuppressive properties, detected in the root and stem extracts of *B. vulgaris* [7].

*Mahonia aquifolium* (Pursh) Nutt. (holly-leaved barberry) also belongs to the Berberidaceae family and is found in eastern Asia, North America, and Central America. The *Mahonia* genus comprises plants with antibacterial, antifungal, and anti-inflammatory properties, widely used in folk medicine as a cure for dysentery, tuberculosis, pharyngolaryngitis, eczema, and other skin disorders [8] due to the high amount of alkaloids [9].

*Phellodendron amurense* Rupr. (Amur cork tree) belongs to Rutaceae family [6] and is among the 50 most important herbs in China [10]. *P. amurense* extract is widely used in traditional medicine for the treatment of pneumonia and diarrhea and for enhancing blood circulation [11], but is also used as a spasmolytic, anti-inflammatory, and as a bitter tonic for stomach problems [12]. An important number of polyphenols, fiber, phytosterols, and carotenoids can be found in *P. amurense* extract. It was shown in several studies that total alkaloids obtained from the cortex of *P. amurense* have a protective effect on stomach ulcer [13]. Other recent studies showed that based on its anti-angiogenic effect, *P. amurense* bark extract has a good antiproliferative effect, and can also be taken into consideration for the development of novel anticancer drugs or angiogenesis-dependent disorders [12].

Several diseases such as diabetes, hormonal disorder, diarrhea, obesity, coronary heart disease, and hyperlipidemia were shown recently to have been effectively inhibited by berberine [14]. Berberine is also known for its antifungal, antiprotozoal, and antimicrobial activities. Furthermore, other studies have shown that berberine has potent anticancer activity towards prostate, colorectal, lung, leukemia, esophageal, glioma, and ovarian cancer cell lines [4,15].

Also, berberine can induce apoptosis of tumor cells by modulating the activity of several pro-apoptotic and anti-apoptotic genes. Thus, it can alter the Bcl-2/Bax ratio and decrease the mitochondrial membrane potential of selected tumor cells [16]. In addition, by activating caspase-3 and caspase-8, and releasing the cytochrome c, berberine might induce apoptosis through the mitochondrial/caspase pathway [17,18]. It was also shown that berberine can affect the cell cycle at lower concentrations, arresting tumor cells in the G1 phase [19]. The arrest of tumor cells in the G2/M phase occurs at higher concentrations of berberine [20]. It was reported that berberine might also induce apoptosis of tumor cells by ROS (reactive oxygen species) generation [21]. Moreover, several reports showed how berberine in combination with radiotherapy or chemotherapy drugs can neutralize their toxicity and enhance therapeutic activities [4].

Melanoma is one of the most common malignancies, with a relatively low response rate to standard anticancer drugs [22]. At the moment, approximately 133,000 new cases of melanoma are diagnosed each year [23]. Treatments for melanoma include surgery, chemotherapy, and immunotherapy. In advanced melanomas, surgical treatment is insufficient, and chemotherapy remains the most commonly used anticancer therapy, especially in the treatment of recurrent and progressive cancers [24].

Lung cancer is a complex and very aggressive disease with multiple mutations, and it has two main subtypes: small-cell lung cancer (15% of all cases) and non-small-cell lung cancer (85% of all cases). Promising new therapies, such as immunotherapy and targeted therapy, have shown relative success in the treatment of lung cancer, due to limitations such as resistance, adverse effects, and high costs, resulting in low survival rates [25].

In vitro studies rely on the so-called two-dimensional (2D) cell models, where standardized cell lines are used to examine the effects of various medicines in flat-bottomed plates or dishes allowing for only limited interaction between the cells and culturing conditions [26]. This is a major limitation since in vitro studies do not reflect the behavior occurring in the in vivo environment. A solution to study fundamental interactions between human cell lines and medicinal compounds is the use of three-dimensional cell cultures that resemble in vivo conditions. This alternative helps to better understand the functionality of culture cells under stress conditions while reducing the costs that might come affiliated to in vivo studies [27].

In cancer theragnostics, recent studies have been focused on targeted therapeutic solutions with minimal negative side effects. The exclusion of certain substances or a change in the way they are used was made possible due to the development of evidence-based medicine [28]. A targeted delivery system usually consists of a nanodevice, to which the desired treatment is applied either by encapsulation or coronation [29]. Owing to several revolutionary developments in nanobiotechnology, the synthesis methods of nanomaterials became less complicated and straightforward, enabling the construction of any type or structure of nanoparticle tailored to essentially every possible application for industry, technology, or medicine [30]. Conventional ways to obtain nanomaterials are based on chemical or physical methods, which often have high-cost production and raise environmental concerns [31]. Green technology is an alternative synthesis technique due to advantages such as low environmental footprint and reduced costs. Among the most studied types of nanoparticles are those based on Zn, Mn, Fe, or noble metals (Pt, Au, or Ag) [32]. In the synthesis of silver nanoparticles (AgNPs), the use of plant sources is economical and the application processes are less complicated [33]. Several studies have shown the benefits as well as the side effects that the use of AgNPs cause in the animal organism. Some of the negative effects are reduced by the use of plant extracts in the synthesis process because of the organic nature of the shell that covers the nanoparticles or by prolonged stabilization of the Ag ions [34,35,36].

This study shows the phytochemical, morphological, and pharmacological characterization of three important berberine-containing plants and how they can be exploited for medicinal purposes. Two cell lines representative of skin melanoma and lung adenocarcinoma were chosen. The plants were first characterized through scanning electron microscopy and the plant extracts were further analyzed through high-performance liquid chromatography. Based on the chemical composition, their potential to inhibit cancer cell development was investigated in 2D and 3D conditions. The efficacy of *B. vulgaris* extract was proven in both in vitro conditions, with the best results against skin melanoma cells. On the other hand, *P. amurense* was shown to have the least antiproliferative effects on both cell lines. These results were consistent with the chemical composition of the plant extracts, where *B. vulgaris* had the highest berberine concentration. Due to these findings, the *B. vulgaris* extract was further used to synthesize AgNPs to investigate the reducing capacities of the plant extract as well. The NPs were characterized only through physical methods, and their pharmacological potential remains to be investigated in future work.

## 2. Materials and Methods

### 2.1. Plant Material

*Berberis vulgaris* L., *Mahonia aquifolium* (Pursh) Nutt., and *Phellodendron amurense* Rupr. were collected from the ‘Alexandru Borza’ Botanical Garden of Cluj-Napoca (46°45′36″ N and 23°35′13″ E) by Dr. M. Parvu, Babes-Bolyai University of Cluj-Napoca. The plants were taxonomically identified and authenticated, and voucher specimens (CL 659 560 for *Berberis vulgaris*, CL 665 978 for *Mahonia aquifolium*, and CL 669 022 for *Phellodendron amurense*) were deposited in the Herbarium of ‘Alexandru Borza’ Botanical Garden, ‘Babeș-Bolyai’ University, Cluj-Napoca, Romania.

### 2.2. SEM Examination

The leaf and stem samples were prepared according to our previous work [37]. Briefly, the leaves and stems were immersed in glutaraldehyde for 1.5 h immediately following harvest. The glutaraldehyde was washed with phosphate-buffered saline and then the samples were dehydrated with alcohol in increasing concentrations. These steps were conducted at 4 °C. The samples were then preserved with hexamethyldisilazane, and after drying, they were covered with a 9 nm layer of platinum–palladium and examined with SEM Hitachi SU8230 (Hitachi, Tokyo, Japan).

### 2.3. Extract Preparation

Fresh stem bark (fragments of 0.5–1 cm) for each species was extracted by cold repercolation method with ethanol (Merck, Bucharest, Romania), at a starting concentration of 70%, at room temperature, for 3 days [5,6,38,39]. The plant extracts were obtained by filtration [39]. So, the *P. amurense* and *B. vulgaris* extracts containing 1 g plant material in 1 mL had 30% final ethanol concentration (*w*/*v*), and the *Mahonia aquifolium* extract 1 g plant material in 1 mL of had 20% ethanol (*w*/*v*).

### 2.4. Plant Extract Characterization

#### 2.4.1. Phytochemical Analysis

The identification and determination of the most important analytes in the studied samples was achieved using an HPLC-DAD approach as indicated in previous work [5,6]. Briefly, the chromatographic separation, identification, and quantification was performed using an Agilent 1200 HPLC system (Waldbronn, Germany) that was equipped with a quaternary pump. An Eclipse XBD-C18 column (150 mm × 4.6 mm, 5 µm particle size) from Agilent (Waldbronn, Germany) was employed and a volume of 10 µL filtered extract was injected. The flow rate was set at 1.0 mL/min and the column was kept at 30 °C. A gradient elution was employed using solvent A, 0.1% TFA, and solvent B as acetonitrile. The gradient was as follows: In the first two minutes, an isocratic step at 5% B; for the interval 2–20 min, a gradient from 5 to 25% B, followed by an isocratic step in interval 20–29 min at 25% B. This isocratic step was followed by a gradient step from 25 to 30% in the interval 29–30 min, and another gradient step from 30% to 100% B in the interval 30–35 min. In the interval 35–37 min, an isocratic step at 100% B was used for washing and in the interval 37–37.5 min quickly back to 5% B, which was kept until 40 min for equilibration. The quantitative determination was determined using external standard calibration that was generated for each compound at five concentrations in the interval 11–340 µg/mL for all the employed standards (gallic acid, 4-hydroxybenzoic acid, caffeic acid, p-coumaric acid, ferulic acid, berbamine, jatrorrhizine, palmatine, and berberine, all of analytical-grade purity for different commercially available sources). The identification of the compounds in the real samples was carried out using the DAD detector (Agilent Technology, Waldbronn, Germany), which measured the entire spectrum in the 190–550 nm region, every 1 s, and the chromatograms were monitored at 280 nm at the specific retention time.

#### 2.4.2. Cytotoxicity Assays

Human skin melanoma (A375, ATCC CRL-1619, Wesel, Germany) and lung adenocarcinoma (A549, CRM-CCL-185, Wesel, Germany) were used to determine the anticancer effect of the plant extracts. The MTT, LDH, and Griess assays were used according to previous work [40]. Thus, the cells were incubated with the extracts in 96-well plates in increasing concentrations, at 10^4^ cells/well confluence. The extracts were left to interact with the cells for 24 h, after which the LDH and NO were measured from the media, and the viability was calculated from cells remaining in the wells. The concentration at which 50% of cells are affected (IC50) was next calculated from the viability assay.

Based on the observed results, the plant extracts were also tested against A375 and A549 3D cell cultures. The spheroids were grown in round-bottomed 96-well plates with ultralow attachment and the confluence was kept at 10^4^ cells/well. After 24 h, the treatment was applied in increasing concentrations and the MTT and LDH biochemical assays were conducted.

#### 2.4.3. Nanoparticle Synthesis

The Ag nanoparticles were obtained according to our previous work [41]. Briefly, 5 mM AgNO_3_ (VWR International GmbH, Wien, Austria) solution was mixed with the plant extract (AgNO_3_ solution: plant extract = 1:2, *v*/*v*) for 5 and 18 h at 1000 rpm and room temperature. The change in color from dark green to brown indicated the nanoparticle (NP) formation. The NPs were washed with water and ethanol by repeated centrifugations at 7000 rpm and dried at 60 °C for 24 h. The morphological examination was conducted using a transmission electron microscope (TEM) Hitachi HD2700 (Hitachi, Tokyo, Japan) coupled with a double EDX detector.

For the X-ray diffraction (XRD) investigation the high-resolution SmartLab X-ray diffractometer (Rigaku, Tokyo, Japan), operated at 9 kW and coupled with SmartLab Guidance software (SmartLab Studio II package software, Rigaku, Tokyo, Japan) was used.

## 3. Results

### 3.1. Plant Characterization

#### 3.1.1. Stems and Leaves of *Berberis vulgaris*, *Mahonia aquifolium*, and *Phellodendron amurense*

**Figure 1:**
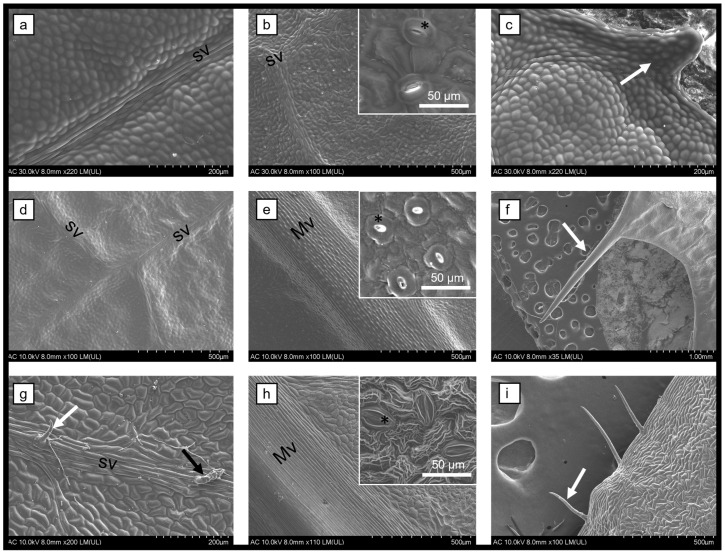
Scanning electron microscopy micrographs of the leaves of *B. vulgaris* (**a**–**c**), *M. aquifolium* (**d**–**f**), and *P. amurense* (**g**–**i**) showing the upper epidermis (**a**,**d**,**g**), the lower epidermis (**b**,**e**,**h**), and the margins (**c**,**f**,**i**); Mv = midvein; sv = secondary vein; white arrow = protuberance/tector trichome; black arrow = secretory trichome; * = stomata.

**Figure 2:**
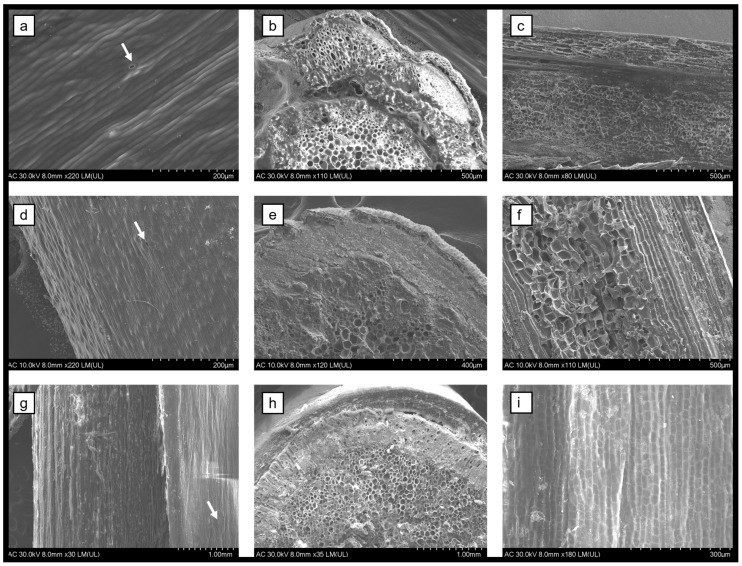
Scanning electron microscopy micrographs of the stems of *B. vulgaris* (**a**–**c**), *M. aquifolium* (**d**–**f**), and *P. amurense* (**g**–**i**) showing the surfaces (**a**,**d**,**g**), the cross-sections (**b**,**e**,**h**), and the longitudinal sections (**c**,**f**,**i**) of the stems with stomata distributed randomly (white arrow) and normal aspect of the vascular bundles.

The stems and leaves of *Berberis vulgaris*, *Mahonia aquifolium*, and *Phellodendron amurense* were investigated through scanning electron microscopy (SEM). The leaves are the site of natural compound synthesis, while the bark is used for storage. The morphological aspect of both organs provides information regarding the general health of the plant. The leaves are hypostomatic and present protuberances or trichomes on the margins (Figure 1). The leaves of *P. amurense* present two types of glandular trichomes (secretory and tector), distributed also on the midvein and secondary veins (on the upper epidermis only), while the other two species have only tector trichomes. The SEM characterization revealed the presence of stomata on the surface of the stem (Figure 2), while the cross-sections and the longitudinal sections showed a normal distribution of cells with no microbial infections.

#### 3.1.2. Chemical Compounds in Plant Extracts

**Figure 3:**
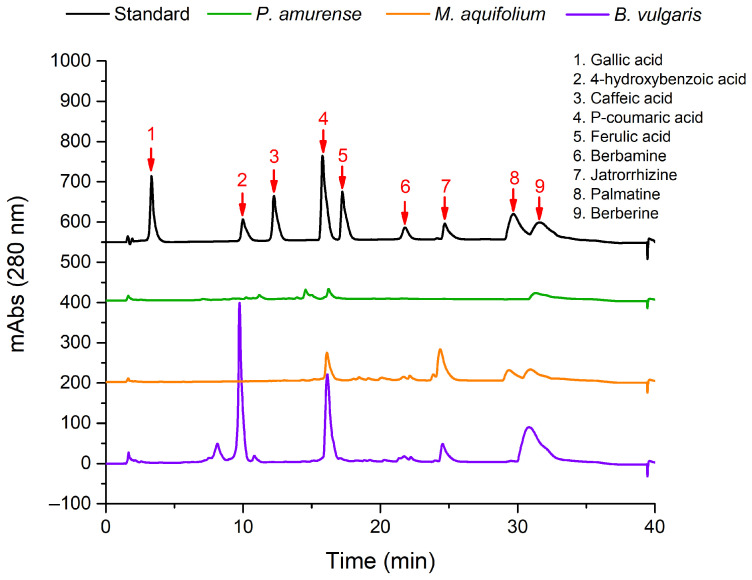
HPLC chromatograms of the *B. vulgaris*, *M. aquifolium*, and *P. amurense* plant extracts monitored at 280 nm; the detailed quantitative data are given in Table 1.

The plant extracts obtained were investigated through high-performance liquid chromatography (HPLC) to determine their chemical composition (Figure 3). Out of the three extracts, *B. vulgaris* and *M. aquifolium* had high levels of phytochemical constituents, unlike *P. amurense*, which was proven to have low levels of the analytical standards tested (Table 1). *B. vulgaris* was richest in berberine (10.2 ± 1.1 mg/g), followed by jatrorrhizine (5.27 ± 0.43 mg/g) and berbamine (1.32 ± 0.11 mg/g), while caffeic, p-coumaric, and ferulic acids were under the limit of detection. However, additional peaks that could be attributed to other natural compounds were observed. For *M. aquifolium*, jatrorrhizine (12.7 ± 1 mg/g) had the highest concentration, and for *P. amurense* it was berberine (2.63 ± 0.22 mg/g), while traces of gallic and 4-hydroxybenzoic acids were found only in *B. vulgaris* and *P. amurense*.

### 3.2. Pharmacological Potential

#### 3.2.1. Cytotoxicity Assays

The pharmacological potential of the plant extracts was tested in vitro on human skin melanoma (A375) and lung adenocarcinoma (A549) cell lines. The viability was determined through the MTT (3-(4,5-dimethylthiazol-2-yl)-2,5-diphenyltetrazolium bromide), the membrane integrity was determined from the media through the lactate dehydrogenase (LDH) assay, and the nitrite/nitrate concentration was determined through the Griess assay.

**Figure 4:**
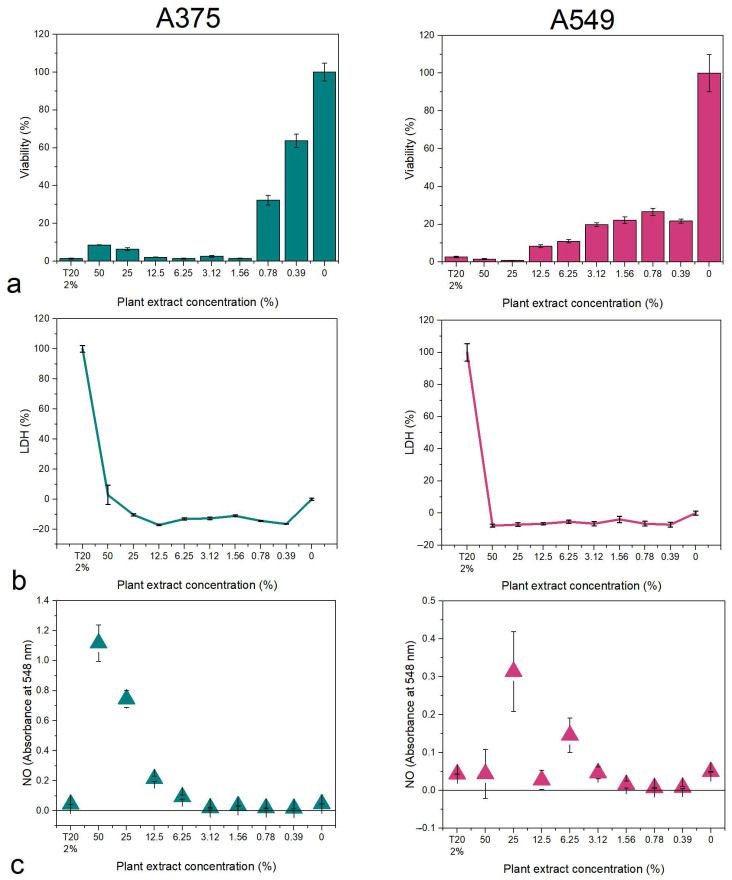
Cytotoxicity of the *B. vulgaris* extract on A375 skin melanoma and A549 lung adenocarcinoma: (**a**) MTT viability assay; (**b**) LDH membrane integrity assay; (**c**) NO Griess assay. T20 = Tween 20 negative control.

The *B. vulgaris* extract reduced the viability of A375 cells starting with the 0.78% concentration of plant extract in the media, while the A549 had low viability values at all tested concentrations (Figure 4a). No LDH release was observed in this case, indicating that the plant extract might induce cell death through apoptosis (Figure 4b). The nitrite/nitrate concentration was high at high concentrations, complementary to the viability assay (Figure 4c).

**Figure 5:**
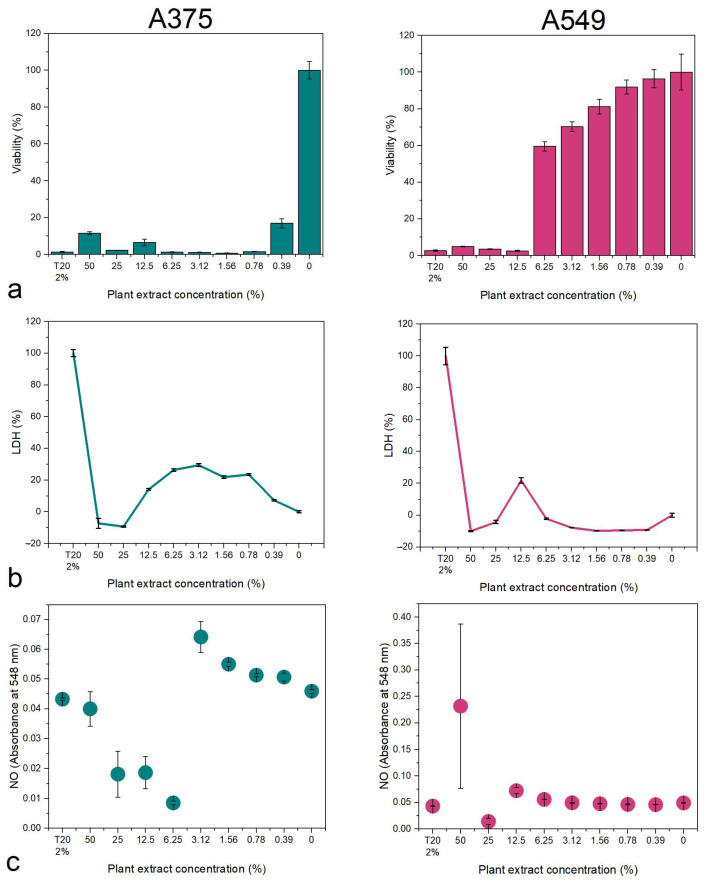
Cytotoxicity of the *M. aquifolium* extract on A375 skin melanoma and A549 lung adenocarcinoma: (**a**) MTT viability assay; (**b**) LDH membrane integrity assay; (**c**) NO Griess assay. T20 = Tween 20 negative control.

The *M. aquifolium* plant extract affected the viability of A375 cells at all tested concentrations, while A549 had a drastic drop in viability value at 12.5% extract concentration (Figure 5a). The LDH release was consistent with the viability results, indicating high values in the case of A375 cells, while for A549 cells, a spike was observed at the 12.5% concentration, and decreased values for the rest (Figure 5b). High values of LDH might indicate cell death through necrosis. The Griess assay revealed levels of nitrites/nitrates consistent with the MTT and LDH assays for both cell lines (Figure 5c).

**Figure 6:**
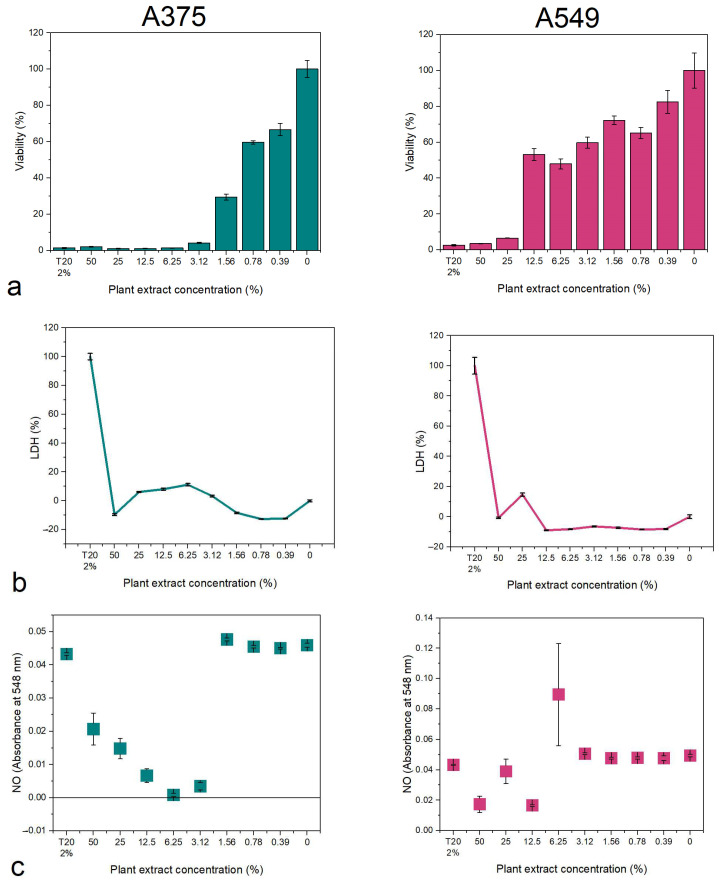
Cytotoxicity of the *P. amurense* extract on A375 skin melanoma and A549 lung adenocarcinoma: (**a**) MTT viability assay; (**b**) LDH membrane integrity assay; (**c**) NO Griess assay. T20 = Tween 20 negative control.

In a similar manner *P. amurense* affected the viability of A375 cells starting with the 1.56% concentration, while for A549, the 25% extract showed a significant decrease in the viability values (Figure 6a). The LDH release was consistent with the MTT assay for both cell lines (Figure 6b), as well as for the Griess assay (Figure 6c).

With the help of the abovementioned assays, the concentrations at which 50% of cells are affected (IC50) were calculated (Table 2). The results showed that *B. vulgaris* has the strongest inhibitory capacity against both cell lines, while *M. aquifolium* has better potential against A375 cell lines, and *P. amurense* was the least toxic of the three tested plant extracts.

**Figure 7:**
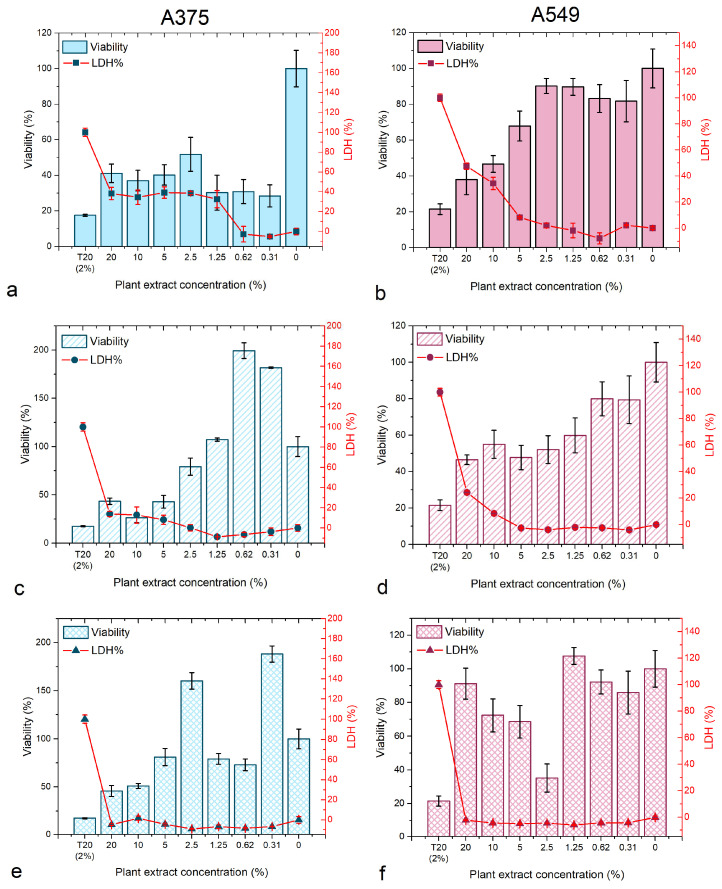
Viability and membrane integrity (LDH% release) of *B. vulgaris* (**a**,**b**), *M. aquifolium* (**c**,**d**), and *P. amurense* (**e**,**f**) plant extracts on A375 and A549 spheroids.

The viability and membrane integrity of the 3D A375 and A549 cell lines was further investigated. Generally, A375 was more sensitive to the plant extracts compared to A549 cells (Figure 7). The most potent in inhibiting the evolution of the spheroids were *B. vulgaris* and *M. aquifolium*, with a dose-dependent reaction where the viability decreased with the increase in concentration. At higher concentrations, the LDH level in the media was relevant to the viability loss, increasing while the viability decreased. No increased LDH levels were observed for low concentrations.

*P. amurense* was the least potent extract against both types of spheroids with no relevant reaction dependent on the dose used.

Using the IC50 values determined for the 2D cell culture experiments, seven concentrations were chosen to investigate the effect of the extracts on the viability and membrane integrity of the 3D cell cultures. The IC50 values were calculated for the spheroids (Table 3) and the results revealed that 3D A375 is most sensitive to the *B. vulgaris* extract (3.79% extract concentration), while the least potent was *P. amurense* against 3D A549 (268.58% extract concentration).

#### 3.2.2. Nanoparticle Synthesis

**Figure 8:**
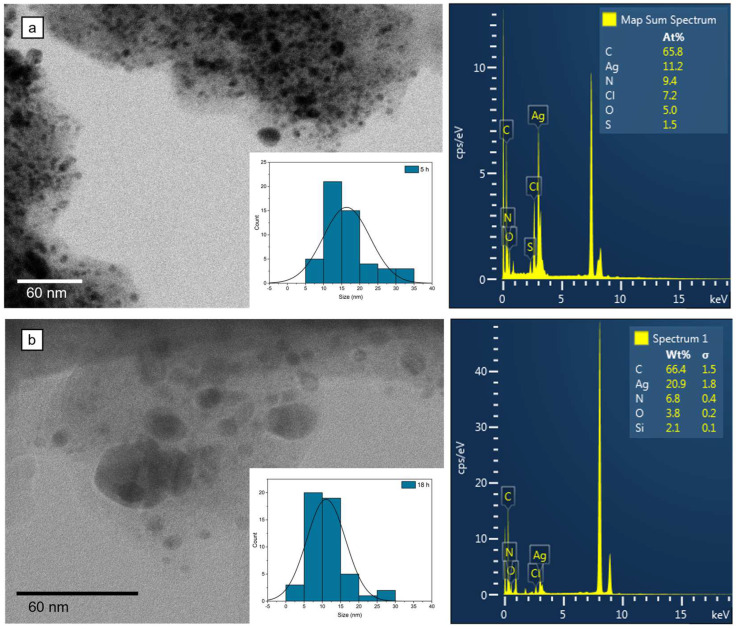
Transmission electron micrographs and EDX spectra of Ag nanoparticles synthesized with *B. vulgaris* plant extract for 5 h (**a**) and 18 h (**b**). It can be observed that nanoparticles can be formed after 5 h of incubation with the plant extract, with a wider size distribution (insets) compared with 18 h of incubation.

The capacity to reduce silver nitrate to silver nanoparticles (NPs) was assayed for the *B. vulgaris* plant extract, at 5 h and 18 h of continuous stirring (Figure 8). This method was implemented to observe whether small and uniformly distributed nanoparticles can be obtained faster for cost efficiency. After 5 h of incubation with the plant extract, uniform spherical nanoparticles were obtained, with an average size of 16.32 ± 6.48 nm (mean ± standard deviation (s.d.); N = 50). After 18 h of incubation, uniformly distributed nanoparticles with an average size of 11.03 ± 5.3 nm (mean ± s.d.; N = 50) were obtained.

**Figure 9:**
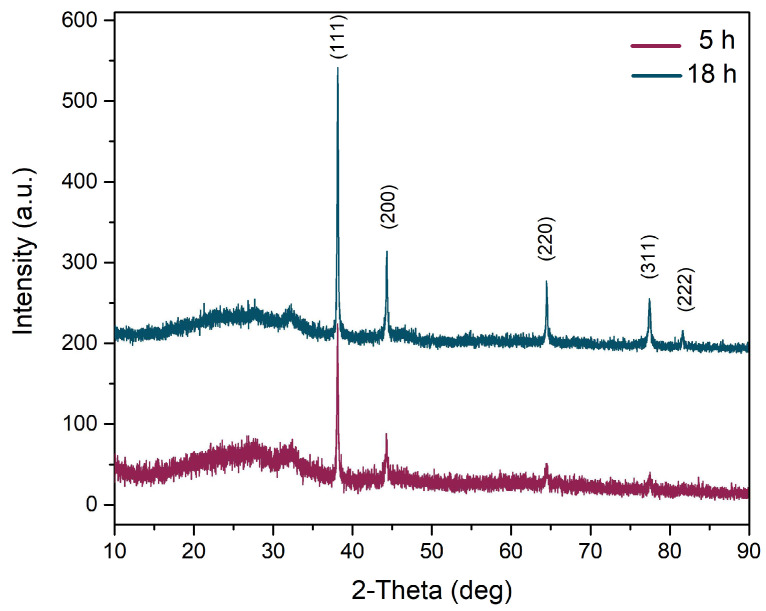
XRD pattern of the resulting Ag nanoparticles.

The crystallinity of the nanoparticles was confirmed by XRD analysis (Figure 9). Both samples had four distinct diffraction peaks at 2θ values that corresponded to the reflection planes of (111), (200), (220), and (311), characteristic of the face-centered cubic structure of silver. However, the samples obtained after 18 h had a distinct peak at (222) as well (reference file PDF card no. 03-065-8428).

## 4. Discussion

The pharmacological potential of three berberine-containing plant extracts was shown in this study. Medicinal plants synthesize and store their natural compounds in the areal parts as a mechanism of protection against pathogens and herbivores. Usually, the place of synthesis is in the leaves, while the bark and stems are used for storage. Previous studies showed how the morphological and anatomical structures of the organs used for extract preparation influence the overall quality of the plant extract and the variability within the species [37,42,43,44]. The specialized epidermal cells (i.e., trichomes and stomata) could provide important information about the natural compounds. For example, glandular and secretory trichomes are used for the biosynthesis, storage, and secretion of phytochemicals [45]. The leaves analyzed herein showed the presence of tector trichomes for *B. vulgaris* and *M. aquifolium*, while *P. amurense* also had secretory trichomes.

The leaves of all three species analyzed herein are hypostomatic. The stomata are responsible for the exchange processes between the plant and the environment. The factors that decide the stomatal index are also responsible for trichomes’ density. Thus, the transpiration process is balanced, and the quality and quantity of natural compounds is influenced [37,45,46,47].

The plant extracts were obtained from the stem of the plants, known to store high quantities of alkaloids, including berberine. According to the HPLC analysis, from all species, *B. vulgaris* had the highest concentration of berberine, followed by *M. aquifolium* and *P. amurense.* Considering that *P. amurense* was the only species with glandular trichomes that are specialized in storing natural products, this could explain why the concentration of berberine and other compounds was low for this species.

Phytoconstituents are responsible for the pharmacological effects of the plant extracts. The whole-plant extract of berberine-containing plants was previously reported to have anticancer, antioxidant, and antimicrobial effects [38,48,49,50,51]. Herein, we showed how skin melanoma and lung adenocarcinoma cell lines were affected by the obtained plant extracts in a dose-dependent manner and at different degrees depending on the plant extract. Studies showed how the *B. vulgaris* root bark extract inhibited the development of human breast adenocarcinoma without affecting normal cells [52]. Damjanović et al. demonstrated the cytotoxic activity of two *M. aquifolium* extracts that inhibited the development of cancerous cells, cell migration, and angiogenesis [53]. Moreover, if the plant extract is combined with conventional cytostatic drugs, the side effects caused by the drug are also reduced [54]. Similar to *B. vulgaris* and *M. aquifolium*, *P. amurense* was also reported to have good anticancer potential [12] and other important pharmacological applications [55,56].

Considering that berberine is a promising anticancer agent [57], this could also explain why *B. vulgaris* showed the best anticancer potential compared to the other two plant extracts obtained herein. Berberine inhibits the proliferation of breast cancer cells by inducing cell cycle arrest [58], promoting apoptosis [59], and enhances LDH release [60]. In colorectal cancer, it was shown that berberine can inhibit the mitochondrial protein synthesis [61], induce cell cycle arrest in G0-G1 phase [62,63], and can also decrease the expression of β-catenin [63,64].

In lung cancer, berberine is able to induce apoptosis through the miR19a/TF/MAPK signaling pathway [16] or the OS/ASK1/JNK pathway [65]; it can inhibit the development by suppressing DNA repair and replication mechanisms [66]. As for skin cancer, berberine also inhibits its proliferation [67]; reduces melanogenesis by the reduction of the phosphorylation of PI3K/AKT, ERK, and GSK3β [68]; and can inhibit the epithelial-to-mesenchymal transition [69]. These and other mechanisms of action might also explain the results obtained herein, for plants containing high berberine concentration [70,71]. The effect of berberine on 3D cancer cell models is insufficiently studied and the current work is a milestone for further studies on spheroid cancer models.

An important biomedical application is the synthesis of metal nanoparticles, using plant extracts. It is important to try and deliver a certain concentration of the plant extract to the site of interest in applications for cancer therapy. Nanoparticles are preferred to achieve this targeted delivery system, and *B. vulgaris* has been previously used to successfully form ZnO [72] or Ag [73,74] NPs. Here, the formation of NPs was shown through TEM, EDX, and XRD analyses. The NPs obtained after 5 h showed additional XRD peaks and other elements that might be attributed to organic components found in the plant extract, which is an indicator that the synthesis process might require additional time. After 18 h of synthesis, the XRD pattern showed a more crystalline structure of the formed AgNPs, with uniform distribution of the size, and less organic matter. These results indicate the reducing capacities of the whole *B. vulgaris* plant extract, and similar results were previously reported [72,73]. *B. vulgaris* can be used to design a nano-targeted delivery system with biomedical applications, in which the addition of chemotherapeutics (i.e., berberine) on the surface is facilitated by the organic shell of the nanomaterials. Further investigations are required to test this theory.

## 5. Conclusions

The morphology of the leaves and stems of *B. vulgaris*, *M. aquifolium*, and *P. amurense* showed features that could explain the differences in the chemical composition and berberine concentration of the obtained plant extracts. The cytotoxicity and potential tumor formation inhibition of the plant extracts were dependent on the plant, cell line, and experimental design, with best results for *B*. *vulgaris*, followed by *M. aquifolium* and *P. amurense*. The reducing capacities as a pharmacological potential was shown with the help of *B. vulgaris*, which resulted in the successful formation of Ag nanoparticles. From this fundamental screening of three berberine-containing plants, *B. vulgaris* was proven most efficient and can be used for further investigations in biomedical applications.

## Figures and Tables

**Table 1:** Quantitative determination of chemical compounds in *Berberis vulgaris*, *Mahonia aquifolium*, and *Phellodendron amurense* plant extracts.

No.	Compounds	Elution Time (min)	*B. vulgaris* (mg/g)	*M. aquifolium* (mg/g)	*P. amurense* (mg/g)
1	Gallic acid	3.32	0.08 ± 0.02	<LOD	0.10 ± 0.01
2	4-hydroxybenzoic acid	9.98	0.26 ± 0.03	<LOD	0.38 ± 0.03
3	Caffeic acid	12.25	<LOD	<LOD	<LOD
4	P-coumaric acid	15.79	<LOD	<LOD	<LOD
5	Ferulic acid	17.24	<LOD	<LOD	<LOD
6	Berbamine	21.80	1.32 ± 0.11	1.09 ± 0.12	<LOD
7	Jatrorrhizine	24.70	5.27 ± 0.43	12.7 ± 1.0	0.37 ± 0.03
8	Palmatine	29.70	0.15 ± 0.02	2.02 ± 0.17	0.09 ± 0.01
9	Berberine	31.59	10.2 ± 1.1	2.84 ± 0.23	2.63 ± 0.22

LOD = limit of detection.

**Table 2:** The concentrations at which 50% of cell lines are affected (IC50) by plant extracts.

Cell Lines	IC50 Values		
	*B. vulgaris*	*M. aquifolium*	*P. amurense*
A375	0.4%	<0.3%	3.5%
A549	0.4%	10.4%	13.8%

**Table 3:** The IC50 values of the spheroids treated with the plant extracts.

Cell Lines	IC50 Values		
	*B. vulgaris*	*M. aquifolium*	*P. amurense*
3D A375	3.79%	12.9%	16.14%
3D A549	13.41%	13.68%	268.58%

## Data Availability

The data are available from the corresponding author upon reasonable request.
